# Mitochondrial Bioenergetics Is Altered in Fibroblasts from Patients with Sporadic Alzheimer's Disease

**DOI:** 10.3389/fnins.2017.00553

**Published:** 2017-10-06

**Authors:** María J. Pérez, Daniela P. Ponce, Cesar Osorio-Fuentealba, Maria I. Behrens, Rodrigo A. Quintanilla

**Affiliations:** ^1^Laboratory of Neurodegenerative Diseases, Universidad Autónoma de Chile, Santiago, Chile; ^2^Centro de Investigación y Estudio del Consumo de Alcohol en Adolescentes, Santiago, Chile; ^3^Instituto de Ciencias Biomédicas, Universidad de Chile, Santiago, Chile; ^4^Departamento Kinesiología, Universidad Metropolitana de Ciencias de la Educación, Ñuñoa, Chile

**Keywords:** mitochondria, Alzheimer's disease, biomarker, OPA1, fibroblasts

## Abstract

The identification of an early biomarker to diagnose Alzheimer's disease (AD) remains a challenge. Neuropathological studies in animal and AD patients have shown that mitochondrial dysfunction is a hallmark of the development of the disease. Current studies suggest the use of peripheral tissues, like skin fibroblasts as a possibility to detect the early pathological alterations present in the AD brain. In this context, we studied mitochondrial function properties (bioenergetics and morphology) in cultured fibroblasts obtained from AD, aged-match and young healthy patients. We observed that AD fibroblasts presented a significant reduction in mitochondrial length with important changes in the expression of proteins that control mitochondrial fusion. Moreover, AD fibroblasts showed a distinct alteration in proteolytic processing of OPA1, a master regulator of mitochondrial fusion, compared to control fibroblasts. Complementary to these changes AD fibroblasts showed a dysfunctional mitochondrial bioenergetics profile that differentiates these cells from aged-matched and young patient fibroblasts. Our findings suggest that the human skin fibroblasts obtained from AD patients could replicate mitochondrial impairment observed in the AD brain. These promising observations suggest that the analysis of mitochondrial bioenergetics could represent a promising strategy to develop new diagnostic methods in peripheral tissues of AD patients.

## Introduction

AD is one of the most common neurodegenerative diseases worldwide and is considered the leading cause of dementia in the world's adult population (Hirtz et al., 2007). From a genetic point of view, AD can be classified into two types, familial cases with autosomal dominant inheritance (FAD), and sporadic AD which primary cause are still unknown (SAD) (Barage and Sonawane, 2015). The accumulation of misfolded proteins such as the amyloid beta peptide (Aß) and hyperphosphorylated tau are considered essential components in the pathogenesis of AD (Querfurth and LaFerla, 2010; Huang and Mucke, 2012). Current evidence suggests that mitochondrial impairment is also a hallmark of AD, as mitochondrial dysfunction appears before the establishment of tau and Aβ pathology contributing to synaptic impairment observed in AD (Knott et al., 2008; Cabezas-Opazo et al., 2015, Yao et al., 2011).

AD mainly affects memory and cognitive functions, and currently, there is an urgent need for an early biomarker with the reliability and accuracy to diagnose this disease. Nowadays, AD diagnosis is based on neuropsychological surveys and in the exclusion of other age-related dementias in consultant patients (Lanfranco et al., 2012). Analysis of this data has determined three stages of AD progression: preclinical AD, mild cognitive impairment type Alzheimer's, and Alzheimer's type dementia (Lanfranco et al., 2012). Unfortunately, the confirmation of AD is only possible during the patient's autopsy observing the characteristic pathological brain lesions (Khan and Alkon, 2015).

Several groups had suggested the use of peripheral tissues as a reliable source of biomarkers that could reflex the metabolic changes in the brain (Yao et al., 2011; Perez et al., 2016). In fact, there are several examples of using skin fibroblasts to study metabolic abnormalities related to neurological (Zoumakis et al., 2007), psychiatric (Kalman et al., 2016), neurometabolic (Khan and Alkon, 2015) and neurodegenerative diseases (Lopez-Erauskin et al., 2012; Ambrosi et al., 2014), in particular Huntington's disease (Marchina et al., 2014), Parkinson's disease (Rakovic et al., 2010; Cooper et al., 2012; Papkovskaia et al., 2012; Mcneill et al., 2013), and genetic forms of Amyotrophic Lateral Sclerosis (Bartolome et al., 2013; Prause et al., 2013; Allen et al., 2014). For that reason, it has been suggested that biochemical changes in brain cells could be reflected in peripheral tissues derived from ectoderm, such as skin (Zoumakis et al., 2007), and that this tissue represents a suitable model to study potential new biomarkers in AD and other neurodegenerative diseases (Khan and Alkon, 2015).

In AD brains, mitochondrial dysfunction is commonly presented as a respiratory failure, with a reduced ATP production, increased reactive oxygen species (ROS) production, abnormal calcium regulation, and mitochondrial fragmentation (Hu et al., 2016). Interestingly, evidence in cortical samples from AD patients suggests an unbalance of mitochondrial fission and fusion, as mitochondrial dynamics is inclined toward fission (Manczak et al., 2011). Therefore, we will evaluate mitochondrial deficits associated with neurodegeneration in fibroblasts of young, age-matched healthy and sporadic Alzheimer's disease patients.

## Methods

### Materials

Chemicals and culture materials were obtained from Sigma-Aldrich (St. Louis, MO, USA), Roche (Alameda, CA, USA), and Invitrogen (Carlsbad, CA, USA). Fluo-3 AM, Thapsigargin, Mitotracker Green FM (MitoGreen), Mitotracker Red CM-H2XRos (MitoRed), FCCP and 2′,7′-DCF were obtained from Molecular Probes (Eugene, OR, USA).

### Patients and cell culture fibroblasts

Skin fibroblasts were obtained from 3 AD patients, three age-matched healthy controls, and one young healthy control, and cultured in growth media containing MEMα (Biological Industries) with 10%FBS (Gibco) and 1% penicillin-streptomycin (Corning) (Vangipuram et al., 2013). The individuals were recruited after providing informed consent approved by the Ethics Committee of the Hospital Clínico de la Universidad de Chile, and Universidad Autónoma de Chile. AD diagnosis was established according to the National Institute of Neurological and Communicative Diseases and Stroke–AD and Related Disorders Association (Mckhann et al., 1984) tests and the Clinical Dementia Rating (CDR) scale (Morris, 1993; Table 1).

**Table 1:** Demographic characteristics of the patients.

**Patient**	**Range age**	**Tests**			**Diagnostic**
		**CDR**	**MoCA**	**MMSE**	
ADP1	80–85	1	19	23	Alzheimer
ADP2	70–75	2	4	2	Alzheimer
ADP3	65–70	3	3	1	Alzheimer
NP1	70–75	0	30	30	Control
NP2	75–80	0	30	30	Control
NP3	80–85	0	25	27	Control
YP	25–30	0	–	–	Control

*The table represents the range of age and diagnosis type for each patient according to the cognitive test applied. The CDR scale indicates the severity of dementia; whereby severe dementia degree (CDR 3), mild-to-moderate dementia (CDR 2), preclinical dementia (CDR 1), and healthy donors (CDR 0). The maximum score for the Mini-mental State Examination (MMSE) and Montreal Cognitive Assessment (MoCA) is 30, with lower scores associated with greater cognitive deterioration*.

### Determination of mitochondrial length

Estimation of mitochondrial length was calculated measuring the length of individual mitochondria along the fibroblasts. Cells were preloaded with MitoGreen (500 nM) at 37°C for 30 min in Krebs-Ringer-HEPES (KRH) buffer supplemented with 5 mM glucose (Quintanilla et al., 2009). The mitochondrial length was classified in: short (>5 μm), medium (5–40 μm) and long (<40 μm) size by the method used in our previous publications (Quintanilla et al., 2009; Hom et al., 2011). We analyzed over 500 individual mitochondria per patient in two independents experiments. For the determination of mitochondrial length following a stimulus, cells previously loaded with MitoGreen were treated with thapsigargin 10 μM. Fluorescence images were taken in an epifluorescence microscopy (Leica LX6000, Germany) using a 63× oil objective. For each independent experiment, we analyzed mitochondrial population from 30 to 40 cells, measuring 25 images for an experiment in each patient, using Image J software.

### Western blot analysis

Cells were lysed in RIPA lysis buffer (AMRESCO) plus protease inhibitor cocktail (Roche). 30 μg of total protein extracts were resolved on a SDS-electrophoresis gel and transferred to nitrocellulose membranes. After blocking process, the membranes were incubated with rabbit polyclonal anti-OPA1 (Thermo Fisher 1:1,000 dilution), rabbit polyclonal anti-DRP1 (Thermo Fisher 1:1,000) and rabbit polyclonal anti-MFN1 (Santa Cruz 1:1,000) antibodies. The equal loading and transfer of membranes were subsequently re-tested with anti-β-actin (Thermo Fisher 1:2,000). After treatment with HRP-linked goat anti-mouse or anti-rabbit secondary antibodies (Thermo Fisher 1:2,000) as indicated, immunoreactive proteins were detected using enhanced chemiluminescence reactive (ECL, Thermo Fisher).

### Mitochondrial membrane potential

The mitochondrial potential was determined using the mitochondrial dye TMRM in non-quenching mode, as described previously with modifications (Quintanilla et al., 2013; Vargas et al., 2014). Mitochondrial potential levels were expressed as the average of fluorescence signal (F) per area in every image minus the intensity of background fluorescence (F0). For determinations in basal conditions, fibroblasts were preloaded with TMRM (100 nM) in KRH-glucose buffer at 37°C for 45 min. Also, to compare mitochondrial potential following a stimulus, we treated the cells previously loaded with TMRM with thapsigargin 10 μM. For each independent experiment, the intensity of the signal was analyzed in more than 30 cells, measuring 25 images per experiment in each patient using Image J software.

### Determination of ROS levels

ROS levels were evaluated using chloromethyl-2,7-dichlorodihydrofluorescein diacetate (DCF) dye (Eugene, OR). Cultured fibroblasts were incubated with 10 μM DCF in KRH-glucose buffer at 37°C for 30 min (Quintanilla et al., 2013). Images were taken adjusting the same exposure time and gain detector to diminish the photobleaching of the dye (Quintanilla et al., 2013). Quantification of fluorescence intensity for each separate experiment was carried out analyzing the intensity of the signal in 25 images for every indicated condition using Image J software. Results are express as the average of fluorescence signal per area in every picture.

### Determination of ATP levels

Total ATP levels were measured in the fibroblasts lysate using a luciferin/luciferase bioluminescence assay kit (ATP Determination Kit #A22066, Molecular Probes, Invitrogen). The amount of ATP in each sample was calculated from standard curves and normalized to the total protein concentration.

### Cytosolic calcium measurements

Cytoplasmic calcium changes were determined using Fluo-3 AM dye. Fibroblasts were loaded with 5 μM Fluo-3 AM in KRH-glucose buffer at 37°C for 30 min (Quintanilla et al., 2013). The signal of the fluorescence background was subtracted from cytosolic cell fluorescence measurements in every experiment, and cytosolic calcium levels were presented as a pseudo-ratio (ΔF/F_0_), as previously described (Quintanilla et al., 2013). To evaluate basal cytosolic calcium levels, we analyzed 25 images for each experiment in every indicated condition. To determinate changes in cytosolic calcium levels following a stimulus, we treated the cells previously loaded with Fluo-3 AM, or in the presence of the mitochondrial uncoupler FCCP (10 μM), or thapsigargin (10 μM), and the changes in fluorescence intensity were registered from 15 cells on average per experiment. Fluorescence intensity and quantification were made using Image J software (NIH).

### Statistical analysis

Student “*t*” test analyzed statistical differences between two group of data. For multiple comparisons, one-way ANOVA was used, followed by Tukey test *a posteriori*. Differences were considered significant if *p* < 0.05, *p* < 0.01, or *p* < 0.001 as indicated.

## Results

### Determination of mitochondrial length in AD fibroblasts

**Figure 1:**
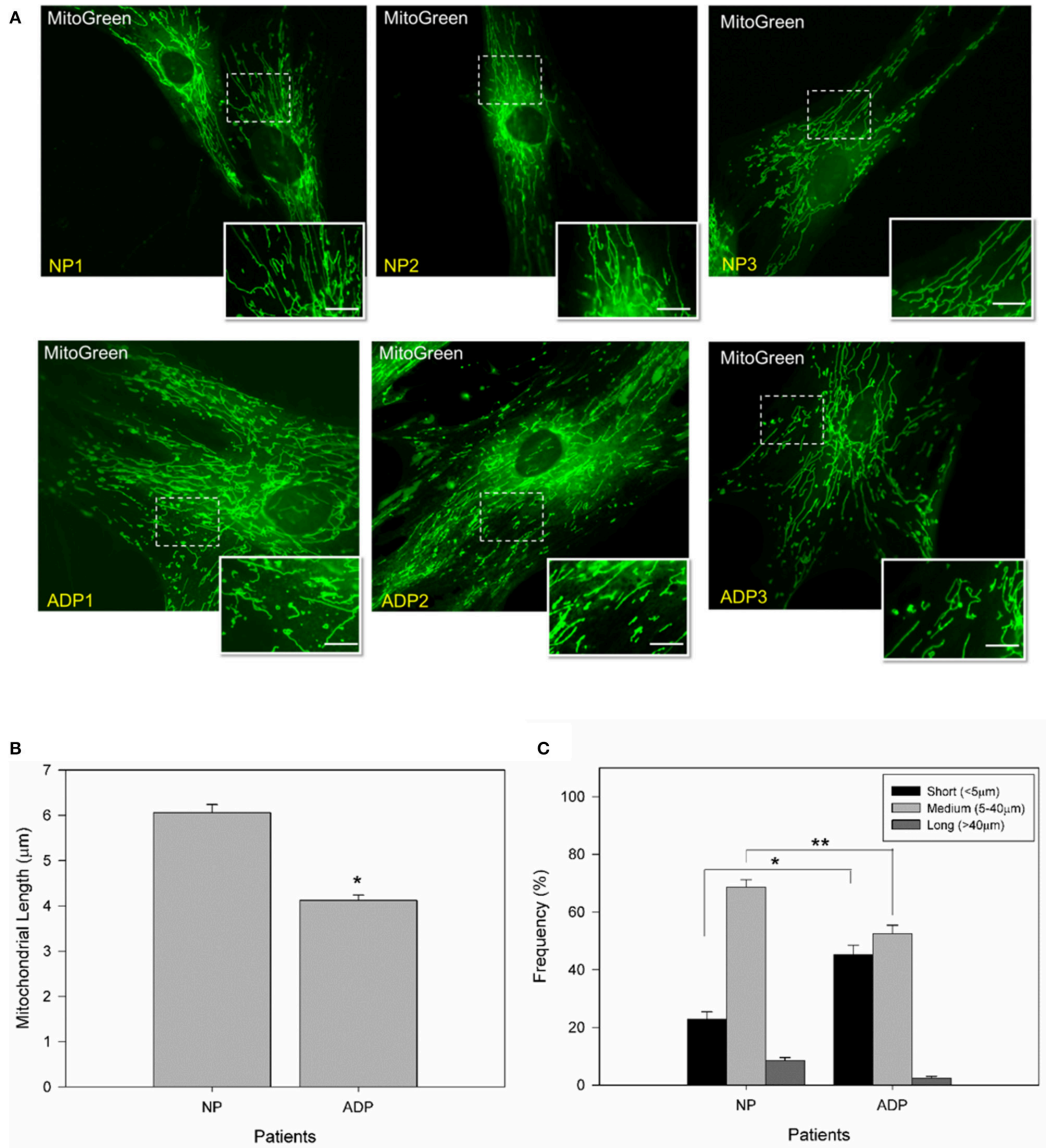
AD cultured fibroblasts present a decrease in mitochondrial length. **(A)** Cells were loaded with MitoGreen to determinate mitochondrial morphology. Bar = 10 μm. **(B)** Quantification of the mitochondrial length average obtained from microscopy analysis (see Methods). Data are mean ± *SE, n* = 3 (technical replicates for each subject). ^*^p < 0.001 indicate differences between groups calculated by Student *t*-test. **(C)** Distribution of mitochondrial length in cultured fibroblasts from AD and control patients. Mitochondrial lengths were grouped in short, medium and long size. Data are mean ± SE, *n* = 3 (technical replicates for each subject). ^*^p < 0.005, ^**^p < 0.003 indicates differences between groups calculated by Student *t*-test between short, medium and long mitochondria from AD and control patients.

To study changes in mitochondrial morphology of sporadic AD fibroblasts, we measured mitochondrial length using MitoGreen in all conditions (Figures 1A–C). AD fibroblasts presented a reduction of 30% in mitochondrial length compared to control patients (Figure 1B). Further classification of the mitochondrial population (see Methods) indicated that AD patients presented 20% more of shorter mitochondria than control patients, with a significant decrease in medium and long size mitochondria compared to control cells (Figure 1C).

### Mitochondrial dynamics is altered in AD fibroblasts

**Figure 2:**
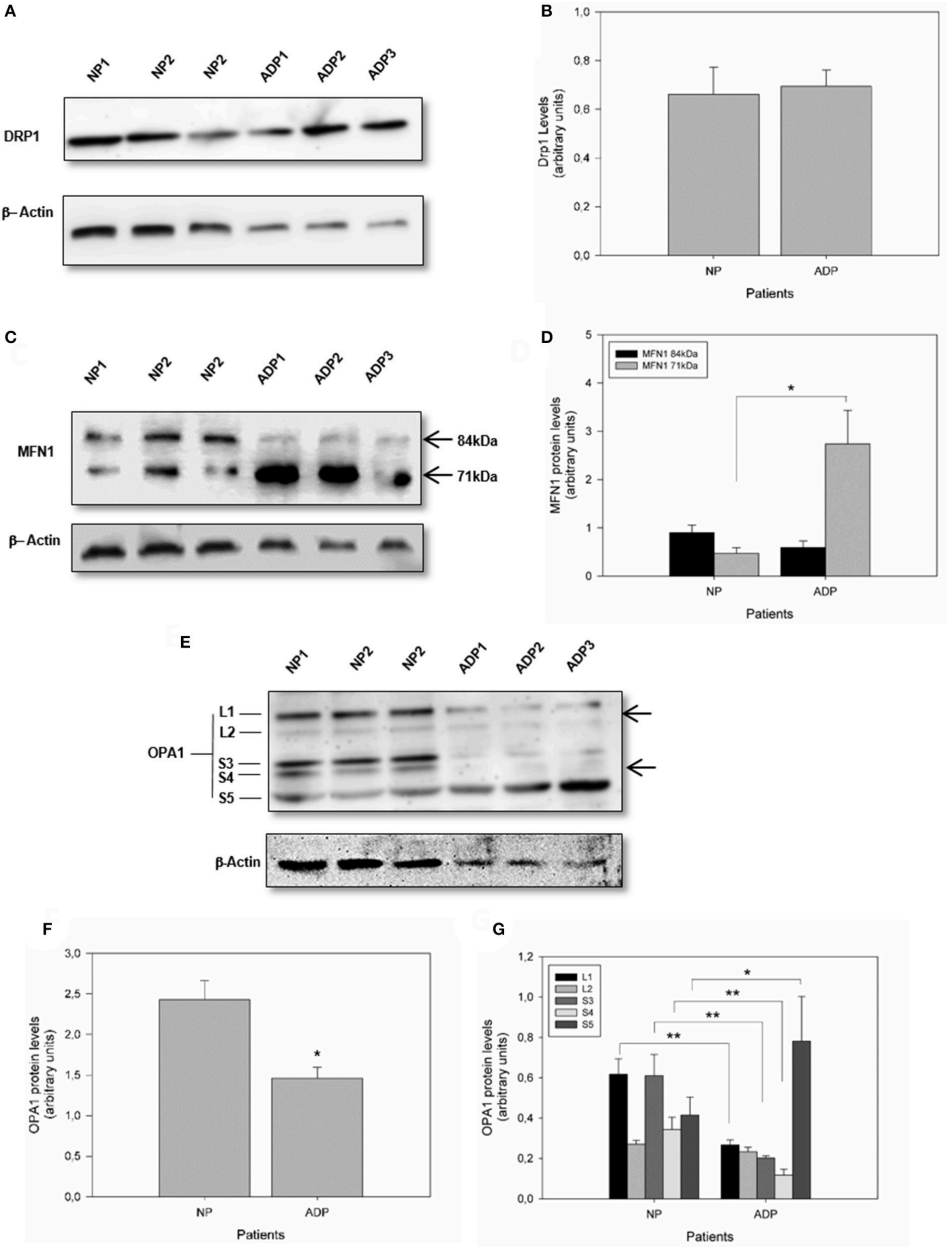
Defects in mitochondrial fusion regulation in fibroblasts obtained from AD patients. **(A)** The levels of mitochondrial fission protein DRP1, in AD and control patients were determined by Western blot (see Methods). **(B)** Quantitative analysis of DRP1 relative expression. Data are mean ± SE. **(C)** Levels of mitochondrial fusion protein MFN1 are indicating the protein splicing variants (arrows). **(D)** Quantification of the MFN1 splicing products. ^*^p < 0.05 indicates differences between groups calculated by Student *t*-test, data are mean ± SE. **(E)** Levels of mitochondrial fusion protein OPA1 showing isoforms and proteolytic products of OPA1 (arrows). **(F,G)** Quantification of OPA1 content and isoforms pattern. ^*^p < 0.05, ^**^p < 0.01 indicate differences between groups calculated by Student *t*-test, data are mean ± SE.

Mitochondrial morphology is modulated by specific large GTPases proteins that control both mitochondrial fission and fusion (Knott et al., 2008). Mitofusins (MFN1 and MFN2) and optic atrophy 1 (OPA1) are proteins required for controlling mitochondrial fusion, and fission depends mainly on dynamin-related protein 1 (DRP1) activity (Wang et al., 2009a; Friedman et al., 2011; Murley et al., 2013). Analysis of Drp1 expression did not show significant differences comparing AD fibroblasts with control patient's cells (Figures 2A,B). Further analysis of MFN1 expression showed a double band at 84kDa and 71kDa in all the samples (Santel et al., 2003) (Figure 2C). Interestingly, in control patients, both splicing variants of the MFN1 protein were equally expressed (Figure 2D). In contrast, AD patients presented a significant increase in the shorter band (Figure 2D). Quantification of OPA1 expression showed a significant reduction in AD fibroblasts respect to the control cells (Figures 2E,F). On the other hand, OPA1 is regulated both at the transcriptional and post-transcriptional level (Rugarli and Langer, 2012), and their processing resulted in the accumulation of two long forms (L1 and L2) and three short soluble forms (S3, S4, and S5) (Rugarli and Langer, 2012). We found that AD fibroblasts showed a specific pattern of OPA1 processing compared to control patients (Figure 2D), which consisted of a significant decrease in L1, S3, and S4 and a prominent increase in S5 form (Figure 2G).

### Defects in mitochondrial bioenergetics in fibroblasts from AD patients

**Figure 3:**
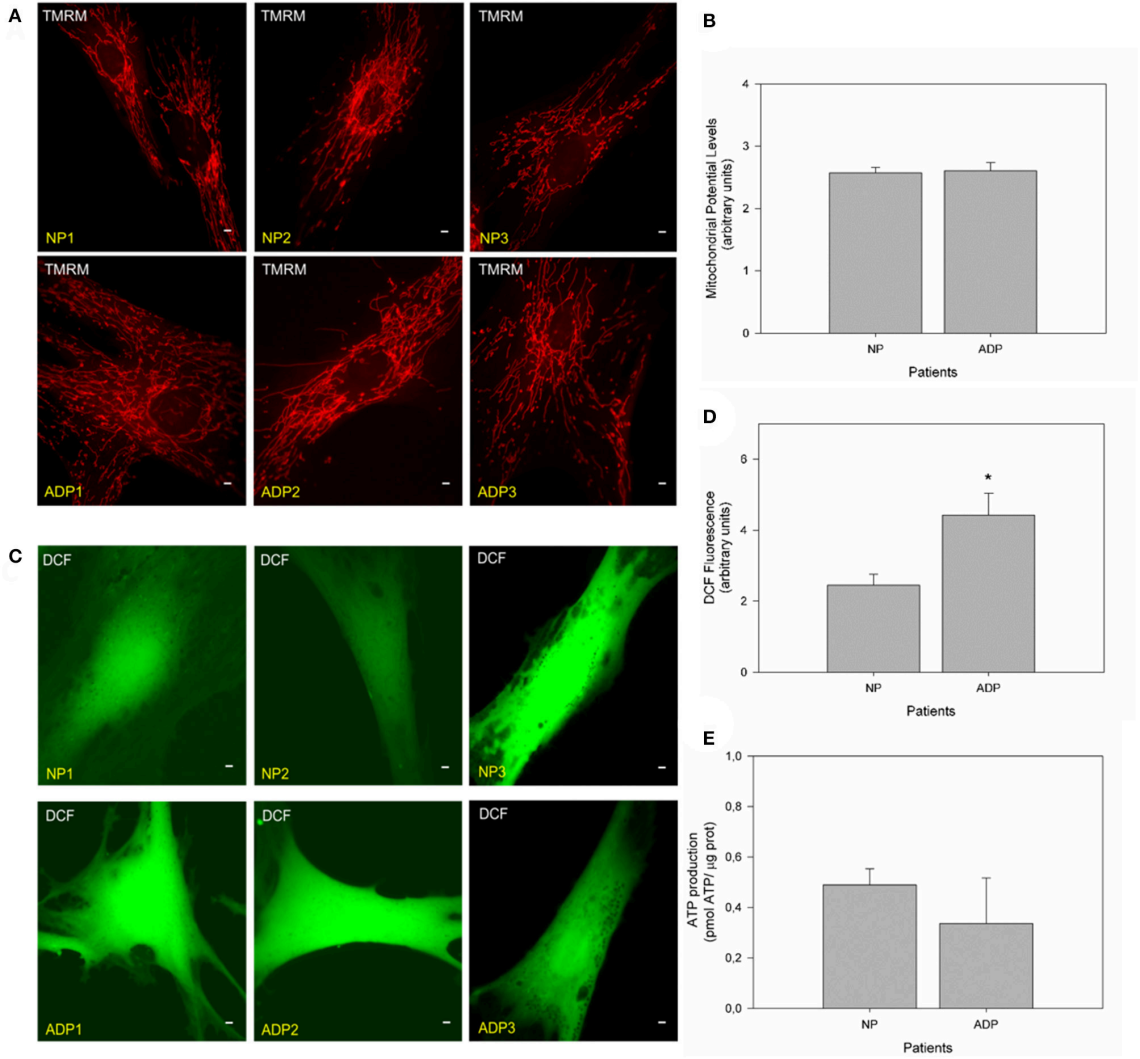
Fibroblasts of AD patients showed an increase in reactive oxygen species (ROS). **(A)** Representative fluorescent images of TMRM show mitochondrial membrane potential levels in fibroblasts of control and AD patients. Bar = 10 μm. **(B)** The graph represents a quantitate data of mitochondrial potential levels as arbitrary units. Data are mean ± SE, *n* = 3 (technical replicates for each subject). **(C)** Representative fluorescent images of DCF intensity. Bar = 10 μm. **(D)** Graph shows quantitative data of ROS levels in each patient's cell type. Data are mean ± SE, *n* = 3 (technical replicates for each subject). ^*^p < 0.05 indicated differences between groups calculated by Student *t*-test. **(E)** The graph shows total ATP levels (pmol) normalized by μg of protein extracted from control and AD fibroblasts. Data are mean ± SE, *n* = 3. ^*^p < 0.05 indicate differences between groups calculated by Student *t*-test.

We evaluated mitochondrial membrane potential in cultured fibroblasts from control and AD patients using TMRM (Figures 3A,B; Brand and Nicholls, 2011; Vargas et al., 2014). No significant changes in mitochondrial potential levels were detected in patients with AD compared to age-matched controls in basal conditions (Figures 3A,B). Complementary, we studied ROS levels using DCF (Quintanilla et al., 2009; Figure 3C). Fluorescence images showed a significant increase in DCF fluorescence levels in AD fibroblasts compared to control cells (Figure 3). Quantitative analysis showed that AD patients showed a significant increase in ROS levels compared to control patients (Figure 3D). We also measured the total ATP levels in all patient fibroblasts (Figure 3E), finding an interesting decrease in ATP levels (Figure S2, ADP2, and ADP3) in AD fibroblasts compared with control cells (NP1-3) (Figure S2). However, further analysis of these data indicated no statistical differences between control and AD patients (Figure 3E).

### Cytosolic calcium defects in fibroblasts from AD patients

**Figure 4:**
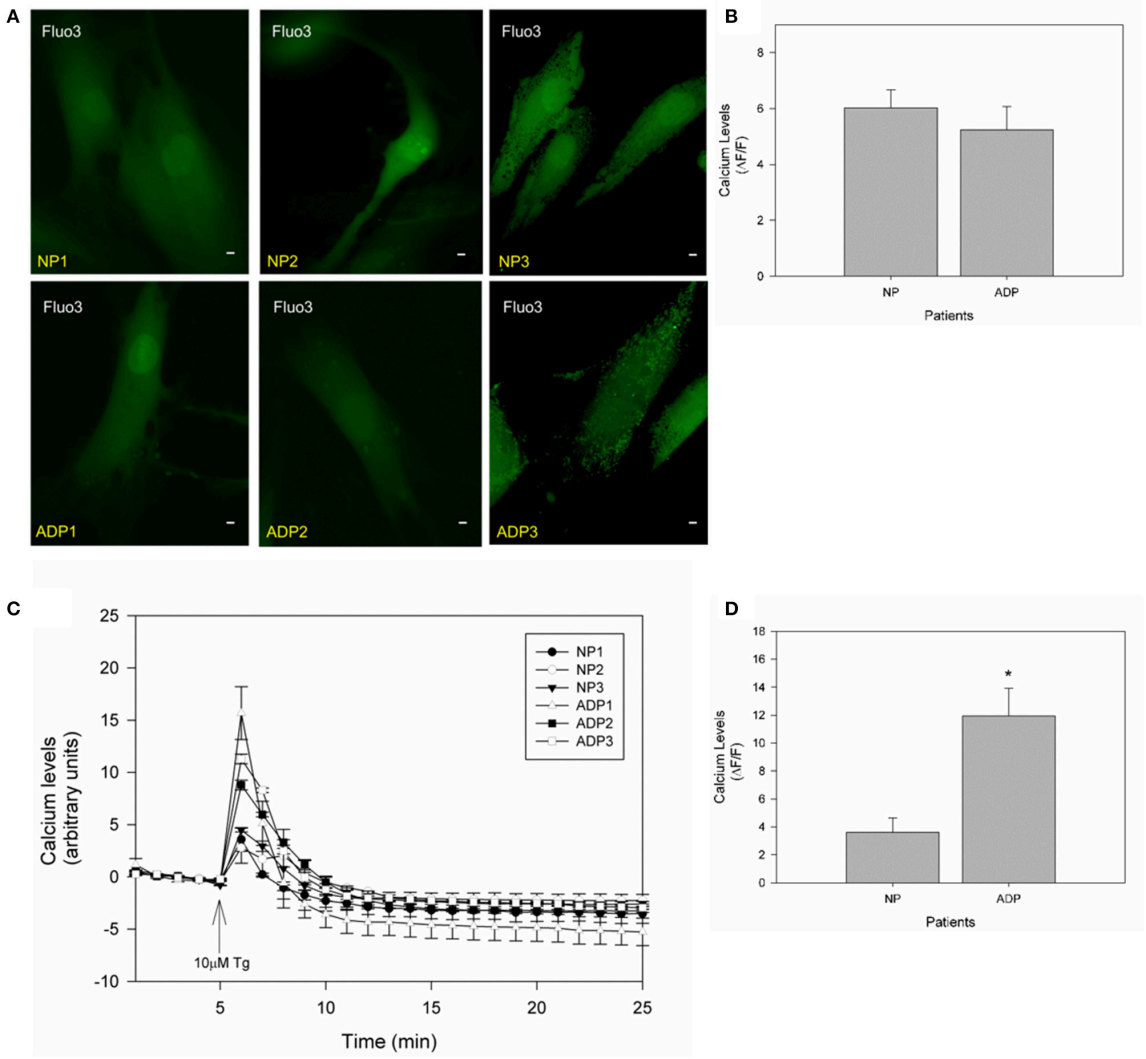
Thapsigargin induces cytosolic calcium handling defects in AD fibroblasts. **(A)** Representative images of Fluo3 intensity showing relative basal cytosolic calcium levels in fibroblasts of control and AD patients. Bar = 10 μm. **(B)** Quantification of relative cytosolic calcium levels as arbitrary units. Data are mean ± SE, *n* = 3 (technical replicates for each subject). **(C)** Representative trends of cytosolic calcium levels over 25 min in AD and control fibroblasts. After 5 min the cells were treated with 10μM of thapsigargin (arrow). Data are mean ± SE, *n* = 3 (technical replicates for each subject). (**D)** Relative cytosolic calcium levels 1 min after treatment with thapsigargin (peak of calcium increase). Data are mean ± SE. ^*^p < 0.05 indicated differences between groups calculated by Student *t*-test.

It is widely accepted that mitochondria have an important role in calcium regulation in physiological and pathological conditions (De Stefani et al., 2016). To study the relative calcium levels in fibroblasts from control and AD patients, we used Fluo-3AM dye, and the cytosolic calcium levels were determined in a fluorescence microscope (Quintanilla et al., 2009). Figure 4A show representative images of relative cytosolic calcium levels in the analyzed fibroblasts, which are showing no significant changes in fluorescence intensity of fibroblasts of AD patients compared to controls (Figure 4B). Further, we evaluated the cytosolic calcium changes in response to a calcium overload stimuli induced by thapsigargin (Quintanilla et al., 2013). Thapsigargin is a cell permeable compound that inhibits calcium uptake by endoplasmic reticulum and thus results in an increase in the levels of intracellular calcium (Quintanilla et al., 2013). We quantified the relative levels of cytosolic calcium after the treatment with 10 μM of thapsigargin for 20 min (Figure 4C). We found that controls and AD fibroblasts present an increase in cytosolic calcium 1 min after the thapsigargin stimulus, but interestingly in all AD patients, this increase was significantly higher than control patients (Figure 4D).

**Figure 5:**
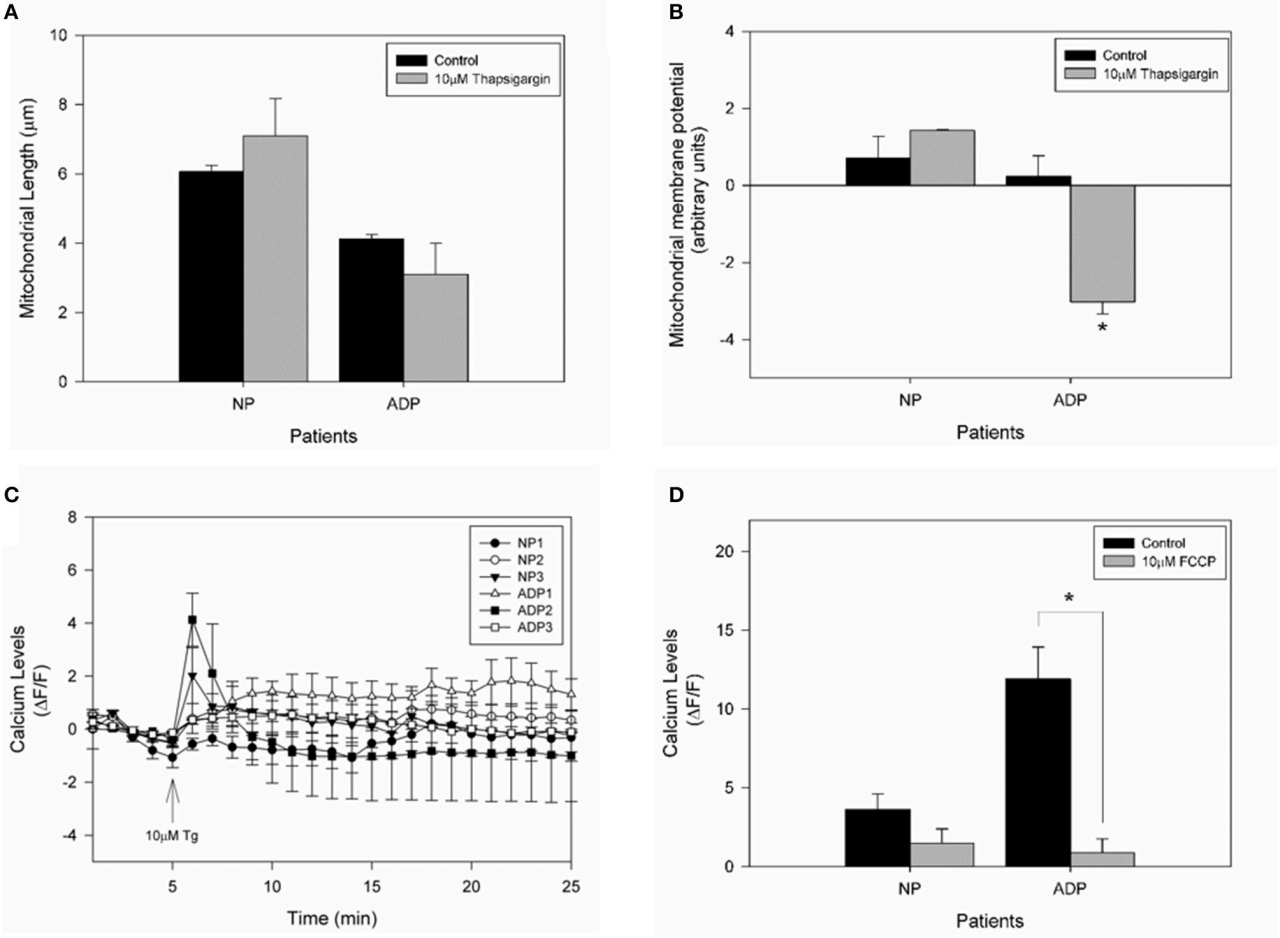
Calcium overload induces mitochondrial impairment in AD fibroblasts. **(A)** Determination of mitochondrial length changes after 1 min treatment with thapsigargin. Data are mean ± SE, *n* = 3 (technical replicates for each subject). **(B)** Mitochondrial membrane potential levels determinate with TMRM of each patient after 10 μM thapsigargin treatment. Data are mean ± SE, *n* = 3 (technical replicates for each subject). ^*^p < 0.05 indicate differences before and after thapsigargin treatment calculated by Student *t*-test. **(C)** Fibroblasts were treated with 10 μM FCCP for 30 min before the experiment was performed. Quantification of the intensity of Fluo3 showing cytosolic calcium levels over 25 min. After 5 min of basal fluorescence detection, the cells were treated with 10μM of thapsigargin (arrow). Data are mean ± SE, *n* = 3 (technical replicates for each subject). **(D)** Quantification of cytosolic calcium levels after 1 min treatment with thapsigargin. Data are mean ± SE. ^*^p < 0.05 indicated differences with or without FCCP, calculated by Student *t*-test.

To assess if calcium stress compromises mitochondrial dynamics, we evaluate mitochondrial length in fibroblasts exposed to thapsigargin (Figure 5). We found that AD fibroblasts presented a reduction in mitochondrial length compared to the untreated condition (Figure 5A). Interestingly, when we studied the changes in mitochondrial potential in response to thapsigargin treatment (Figure 5B), we observed that AD fibroblasts presented a significant decrease in mitochondrial potential (Figure 5B). Finally, to evaluate the contribution of mitochondria in the calcium deregulation observed in AD fibroblasts, we pre-treated cultured fibroblasts with the mitochondrial uncoupler, FCCP, which completely reduces mitochondrial membrane potential (Quintanilla et al., 2013; Figure 5C). We found that in this case, both controls and AD patients presented an equal increase in cytosolic calcium after thapsigargin stimulus (Figure 5C), but that increase is lower than in control condition (Figure 5D). Indeed, AD patients have a significant reduction in cytosolic calcium levels compared to control cells (Figure 5D), suggesting the participation of the mitochondria in calcium dysregulation induced by thapsigargin in AD fibroblasts.

### Fibroblasts of young healthy patients present a characteristic pattern of mitochondrial dynamics and bioenergetics

**Figure 6:**
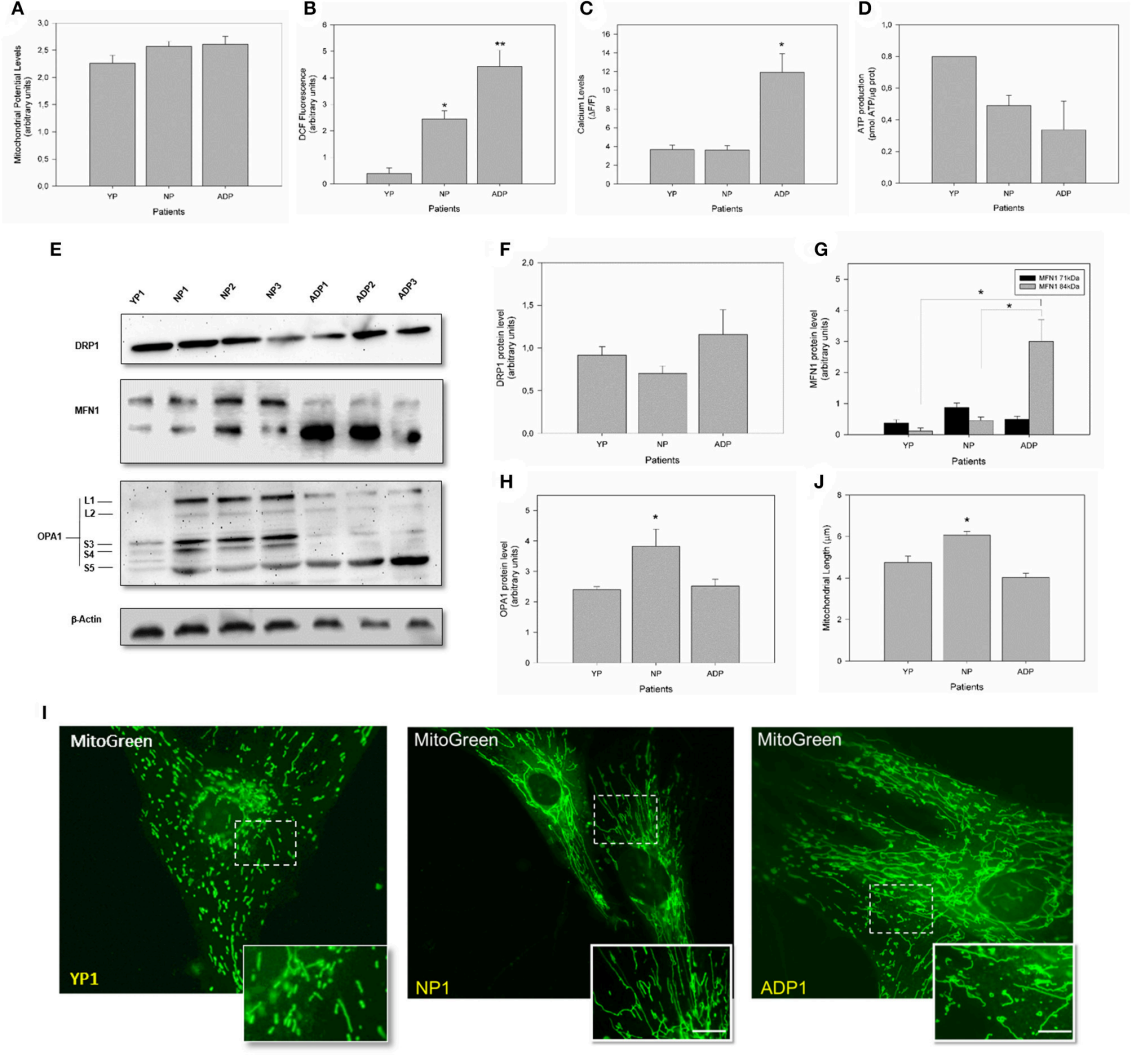
Comparison of mitochondrial bioenergetics profile in young, healthy-aged and AD patient fibroblasts. **(A)** The graph represents the mitochondrial membrane potential levels as arbitrary units. Data are mean ± SE, *n* = 3 (technical replicates for each subject). **(B)** Quantification of total ROS levels in each patient as arbitrary units. Data are mean ± SE, *n* = 3 (technical replicates for each subject). ^*^, ^**^ *p* < 0.05 indicated differences with between groups calculated by one-way ANOVA test. **(C)** Cytosolic calcium levels after 1 min treatment with thapsigargin. Data are mean ± SE. ^*^p < 0.05 indicated differences between groups calculated by one-way ANOVA test. **(D)** Total ATP levels (pmol) normalized by μg of protein. Data are mean ± SE, *n* = 3 (technical replicates for each subject). **(E)** Changes in the protein expression levels of DRP1, MFN1, and OPA1 were assessed by Western Blot in cell extracts from young, aged-match healthy and AD fibroblasts. A representative image of actin western blot is show as internal control (see additional data in Figure S1). **(F)** Quantitative analysis for DRP1 expression levels. Data are mean ± SE. **(G)** Quantitative data indicates splicing products for MFN1 expression. ^*^p < 0.05 indicates differences between groups calculated by one-way ANOVA test, data are mean ± SE. **(H)** Quantification shows total OPA1 expression. ^*^p < 0.05 indicate differences calculated by one-way ANOVA test, data are mean ± SE. **(I)** The mitochondrial length was visualized using a MitoGreen. Bar = 10 μm. **(J)** Measurement of mitochondrial length (average) in all cell groups. Data are mean ± standard error (SE), *n* = 3 (technical replicates for each subject). ^*^p < 0.05 indicate differences between groups calculated by one-way ANOVA test.

To complement our previous observations regarding mitochondrial bioenergetics, we evaluate mitochondrial dynamics and bioenergetics in fibroblasts obtained from young healthy individuals (Figure 6). Mitochondrial potential levels in young fibroblasts did not present significant differences between young, AD patients and age-matched controls (Figure 6A). Interestingly, young fibroblasts showed reduced levels of ROS compared to control cells and AD patients (Figure 6B). When we evaluate the cytosolic calcium response in young fibroblasts, we found a cytosolic calcium peak 1 min after the thapsigargin stimulus. This calcium peak is in the same levels of control cells but decreased compares to AD patients (Figure 6C). Also, young fibroblasts showed increased ATP levels compared to healthy aged and AD patients (Figure 6D).

Analysis of mitochondrial dynamics in young fibroblast showed no differences in DRP1 expression (Figure 6F). However, analysis of MFN1 in young fibroblasts revealed that both splice variants of the MFN1 protein were equally expressed as well as in age-matched controls. However, 71 kDa band is decreased in young patients compared to AD patients (Figure 6G). Surprisingly, we found a significant reduction in the expression of OPA1 in young fibroblasts respect to the old control patients (Figure 6H). Finally, we measured mitochondrial length using MitoGreen (Figure 6I), and young fibroblasts presented a reduction of 15% in the average mitochondrial length compared to control patients with no differences when we compared to AD patient's cells (Figure 6J).

## Discussion

In this work, we evaluated changes in mitochondrial bioenergetics and morphology to identify possible differences in mitochondrial function of fibroblasts from sporadic AD and match-aged healthy patients. Evaluation of mitochondrial bioenergetics showed that AD patients fibroblasts presented higher levels of ROS (Figure 3D) and with no differences in the basal levels of mitochondrial membrane potential (Figure 3A). These data are in agreement with other groups that had shown that AD fibroblasts presented higher levels of basal ROS compared to non-AD patients (Gibson et al., 1996; Naderi et al., 2006; Murphy and Steenbergen, 2011). Also, further analysis of total ATP levels for groups (Control vs. AD group) did not show differences between control and AD patients (Figure 3E). However, the analysis of each patient (Figure S2) indicated that mild-to-moderate and severe AD patients had decreased levels of ATP compared to age-matched healthy patients and preclinical AD patient, suggesting that the compromised bioenergetics state in AD fibroblasts could be related to the progression of the disease.

On the other hand, studies in AD cell models have been shown that mitochondrial potential is only affected under stressor conditions such as calcium or amyloid beta peptide (Quintanilla et al., 2012; Vargas et al., 2014). In that context, we found that in all analyzed patients showed equal levels of basal calcium (Figures 4A,B), and after treatment with thapsigargin produced a significant increase in relative calcium levels in AD patients respect to control cells (Figure 4). Also, thapsigargin treatment induced a further decrease in mitochondrial length and a significant reduction in mitochondrial potential in AD patients (Figures 5A,B), indicating a reduced capacity of the mitochondria to respond to calcium stress. In fact, treatment with the mitochondrial uncoupler, FCCP completely reduced the enhanced calcium increase in AD fibroblasts (Figures 5C,D), suggesting that the incapacity of calcium buffering is related to mitochondrial impairment in AD patients. Interestingly, studies in young healthy patients revealed that young fibroblasts presented higher ATP levels, less ROS production, and intact calcium buffering capacity compared to healthy elderly patients (Figure 6). These findings suggest that in these fibroblasts the aging process could be associated with a reduction of mitochondrial bioenergetics similar to what is observed in mice models (Brunk and Terman, 2002; Lores-Arnaiz et al., 2016; Wyss-Coray, 2016).

Studies in AD brain samples have shown fragmented mitochondria (Wang et al., 2009b), a decrease in mitochondrial density in synaptic structures (Du et al., 2010), and an altered expression of fission and fusion proteins which could explain the mitochondrial fragmentation (Wang et al., 2009a). Here we showed that fibroblasts of sporadic AD patients presented a significant decrease in mitochondrial length and an increase in the percentage of shorter mitochondria respect to the control patients (Figure 1). However, we did not detect differences in the levels of the fission protein DRP1 (Figure 2). These results are opposite to those described previously that shows that levels of DRP1 were decreased in sporadic AD fibroblasts and that was possibly related to an increase in elongated mitochondria accumulated in perinuclear areas of the cells (Wang et al., 2008). However, our results are in agreement with findings presented in AD mice models and cortical AD samples that showed mitochondrial fragmentation (Wang et al., 2008; Cai and Tammineni, 2016), accompanied with an increase in DRP1 (Manczak et al., 2011) and a decrease in total OPA1 levels (Manczak et al., 2011).

Moreover, our results strongly suggest that AD fibroblasts present a specific deregulation of mitochondrial fusion proteins through the reduction of OPA1 and MFN1 expression (Figure 2). In AD fibroblasts the expression of the 84 kDa isoform of MFN1 is decreased and the 71 kDa isoform is significantly increased (Figure 2). Interestingly, it was described that cortical samples of AD patients showed a reduction in the 84 kDa isoform which is related to the severity of the disease (Manczak et al., 2011), but the 71kDa isoform is not present in all the patient's brains (Manczak et al., 2011). Complementary studies showed that the expression of a MFN1-splice variant was specifically upregulated in tumorigenic cells (Chung et al., 2001), suggesting that this different pattern could be related to a disease status (Chung et al., 2001). On the other hand, AD fibroblasts presented a decrease in the expression of total OPA1 and a specific pattern of proteolytic processing, consisting of a decrease in L1, L2, S3, and S4 and an increase in S5 forms (Figure 2). Interestingly, it was described that mitochondrial stress affects the distribution of OPA1 and trigger the complete conversion of L-OPA1 into S-OPA1, leading to a stimulation of mitochondrial fission (Anand et al., 2014). Indeed, this specific increase of the S5 form was described as a result of OPA1 processing induced by H_2_O_2_ (Anand et al., 2014). These are a novel an interesting findings that suggest that mitochondrial fragmentation in AD fibroblasts could be mediated by an increase in MFN1 71kDa isoform and the irregular processing of OPA1.

According to recent publications, the use of skin-derived cells to investigate neurodegenerative diseases with the aim of identifying early biomarkers had to attract increased interest (Clos et al., 2012; Ambrosi et al., 2014; Khan and Alkon, 2015). Indeed, several groups have used use fibroblasts as a model to investigate the mechanisms underlying the pathology of these diseases, with the idea of describing how this cell type present the molecular and metabolic defects that are typically reported at central level (Squitieri et al., 2010; Zanellati et al., 2015; Lopez-Fabuel et al., 2017). For example, Squitieri and collaborators had shown that fibroblasts of Huntington disease's (HD) patients provide a potential peripheral marker as skin biopsies presented pathological mitochondrial features that may predict neuronal pathology (Squitieri et al., 2010). In Parkinson disease (PD), several studies had shown that patient's fibroblasts may be a reliable model system to study mitochondrial dysfunction (Mortiboys et al., 2008; Grunewald et al., 2010; Pacelli et al., 2011; Van Der Merwe et al., 2014), as this cell type present alterations in mitochondrial bioenergetics (Zanellati et al., 2015; Lopez-Fabuel et al., 2017), dynamics (Zanellati et al., 2015), and in the expression and assembly of mitochondrial complexes (Lopez-Fabuel et al., 2017). All these alterations were largely confirmed in mitochondria of cultured neurons and forebrain samples of several PD mouse models (Mortiboys et al., 2008; Grunewald et al., 2010; Pacelli et al., 2011; Van Der Merwe et al., 2014; Lopez-Fabuel et al., 2017). In the case of AD, several studies had shown that fibroblasts of patients with FAD presented a reduction in the mitochondrial number and respiratory function (Gray and Quinn, 2015), high levels of ROS (Richardson, 1993; Moreira et al., 2007), reduced levels of antioxidant defenses (Cecchi et al., 2002), and mitochondrial calcium handling defects (Richardson, 1993; Ito et al., 1994); Therefore, our results presented here indicate that fibroblasts obtained from SAD patients could also present the mitochondrial dysfunction features observed in the AD brain.

Despite the fact that our observations correspond to a limited number of samples, we consider these findings novel and they suggest that mitochondrial function is significantly compromised in AD fibroblasts, presenting identical alterations showed in neuronal cells during AD progression. From our part, further studies will be made increasing the number of samples from control, AD, and MCI patients to corroborate our observations regarding defects in mitochondrial dynamics and bioenergetics.

## Conclusions

AD is a disease commonly associated with cerebral pathology. However, it is accepted that several neurodegenerative diseases where mitochondrial dysfunction is compromised as HD, PD, and ALS, also present also a systemic component that affects peripheral tissues outside the central nervous system. The possibility that skin fibroblasts could reflect these metabolic changes produced in the brain makes this cell type a suitable model to study new biomarkers of AD.

Despite the small group of samples, we were able to analyze several changes that are characteristic of mitochondrial damage. Interestingly, fibroblasts obtained from patients with sporadic AD show specific defects in mitochondrial dynamics and bioenergetics suggesting a new possible target in search of peripheral biomarkers for AD.

To our knowledge, the present study represents an interesting characterization of the mitochondrial status of patient's fibroblasts and suggests that the evaluation of mitochondrial function could differentiate normal aging of pathological AD aging. Our work opens the possibility of a new target for the development of AD biomarkers and presents a novel strategy for epidemiological studies in this disease.

## Ethics statement

A total of 7 patients participated in this study after signed an informed consent approved by the Bioethics Committee of the Hospital Clínico de la Universidad de Chile. In severe cases of dementia, their caregivers provided the consent. This study as been endorsed by the Bioethics Committee of the Hospital Clínico de la Universidad de Chile and validated by the Universidad Autónoma de Chile.

## Author contributions

MP: Performed research, designed experiments, collected data, analyzed data, wrote paper. DP: Collected data, analyzed data. CO: Collected data, revised paper. MB: Contributed with patients samples, analyzed data, revised paper. RQ: Directed the project, designed experiments, contributed important reagents, analyzed data, and revised paper.

### Conflict of interest statement

The authors declare that the research was conducted in the absence of any commercial or financial relationships that could be construed as a potential conflict of interest.

## Supplementary material

Figure S1 Mitochondrial fusion is defective in fibroblasts obtained from AD patients. Figure shows full western blot membranes and all loading controls for mitochondrial dynamic proteins expression. **(A)** The levels of mitochondrial fission protein DRP1, and their internal control actin. **(B)** Levels of mitochondrial fusion protein MFN1, and their internal control actin. **(C)** Levels of mitochondrial fusion protein OPA1, and their internal control actin.

Figure S2 AD fibroblasts present reduced ATP levels compared to age-healthy cells. The graph shows total ATP levels (pmol) normalized by μg of protein extracted from each control and AD fibroblasts. Data are mean ± SE, *n* = 3 (technical replicates for each subject). ^*^*p* < 0.05 indicate differences between groups calculated by one-way ANOVA.

## Abbreviations

AD: Alzheimer's disease
ADP: Alzheimer's disease patient
CDR: Clinical Dementia Rating
DCF: chloromethyl-2,7-dichlorodihydrofluorescein diacetate
DRP1: Dynamin-related protein 1
FCCP: Carbonyl cyanide-p-trifluoromethoxyphenylhydrazone
GTPase: Guanosine triphosphatase
HD: Huntington Disease
NP: Normal patient
MFN: Mitofusin
MMSE: Mini-mental State Examination
MoCA: Montreal Cognitive Assessment
OPA1: Optic atrophy 1 protein
PD: Parkinson Disease
ROS: Reactive oxygen species
TMRM: Tetramethyl-rhodamine methyl ester.
