# Supplementary figures and images for: Mitochondrial Bioenergetics Is Altered in Fibroblasts from Patients with Sporadic Alzheimer's Disease
Source: Front Neurosci. 2017 Oct 6;11:553. doi: 10.3389/fnins.2017.00553 (PMC5635042; doi:10.3389/fnins.2017.00553)

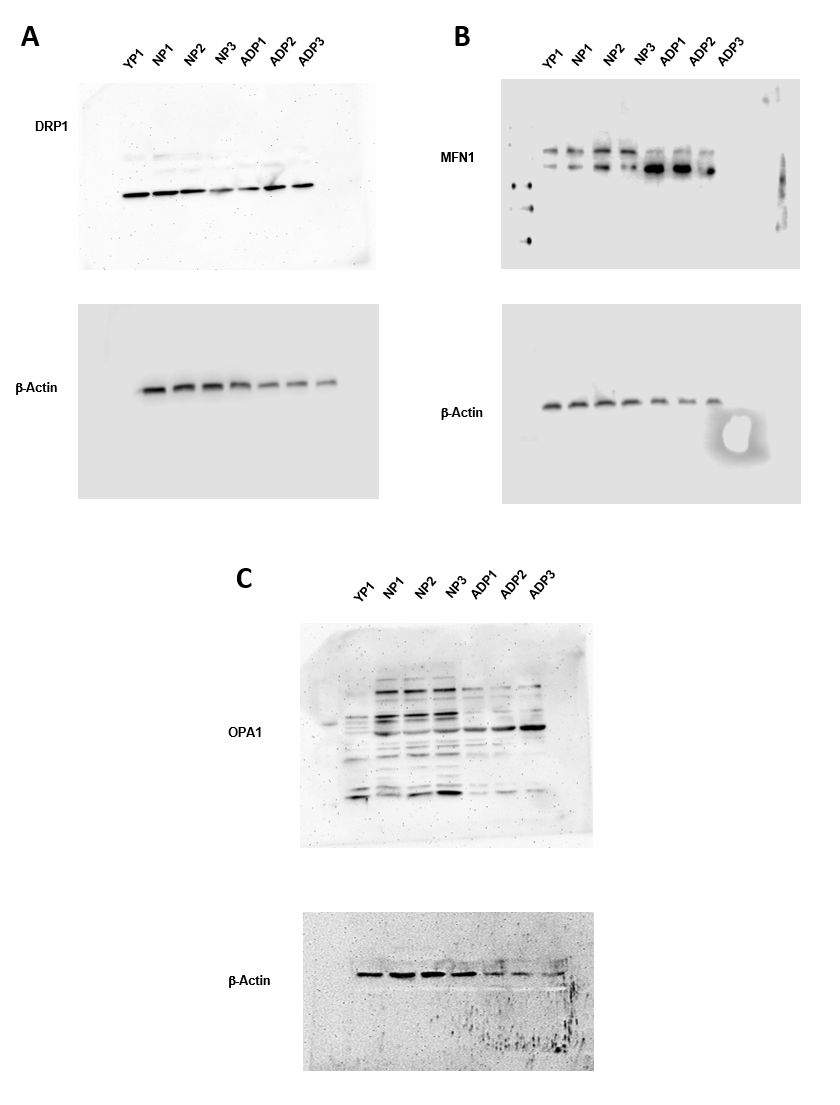

Supplement: Figure S1 — Mitochondrial fusion is defective in fibroblasts obtained from AD patients. Figure shows full western blot membranes and all loading controls for mitochondrial dynamic proteins expression. (A) The levels of mitochondrial fission protein DRP1, and their internal control actin. (B) Levels of mitochondrial fusion protein MFN1, and their internal control actin. (C) Levels of mitochondrial fusion protein OPA1, and their internal control actin. [file Image1.TIF]

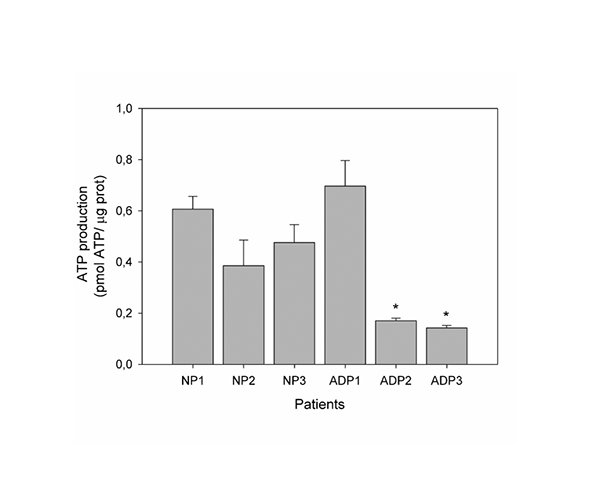

Supplement: Figure S2 — AD fibroblasts present reduced ATP levels compared to age-healthy cells. The graph shows total ATP levels (pmol) normalized by μg of protein extracted from each control and AD fibroblasts. Data are mean ± SE, n = 3 (technical replicates for each subject). *p < 0.05 indicate differences between groups calculated by one-way ANOVA. [file Image2.TIF]
